# CGRP and migraine

**DOI:** 10.1007/s00415-015-8000-4

**Published:** 2016-01-08

**Authors:** M. Cauchi, N. P. Robertson

**Affiliations:** Department of Neurology, Institute of Psychological Medicine and Clinical Neuroscience, Cardiff University, Cardiff, UK

Migraine has been estimated to be the seventh highest cause of disability worldwide, and the third most common disease worldwide after dental caries and tension type headache. However, the use of currently available acute and prophylactic medications to control this condition, such as 5-HT1 agonists (triptans) and beta-blockers, is limited by side effects and efficacy so that alternative and more specific treatments are required. More recently, an improved understanding of the pathophysiology of disease has allowed investigation of new therapeutic targets.

The 37 amino acid neuropeptide calcitonin gene-related peptide (CGRP) has been shown to play a crucial role in the trigeminocervical complex pathway for nociception in the head. Studies have demonstrated elevated levels in the external jugular vein during the headache phase of migraine, with reduction following headache resolution. Furthermore, CGRP infusion triggers migraine type headache and subsequent treatment with triptans results in normalization of CGRP levels. This neuropeptide is therefore thought to have a central role in pain modulation as it participates in the neurovascular pathway and contributes to the vasodilation and neurogenic inflammation, which leads to migrainous attacks. Targeting CGRP may provide the ideal therapeutic tool needed for control of this common and debilitating illness.

The three studies chosen for this month’s journal club are a small sample of the large amount of research being performed on CGRP. The first investigates whether its measurement can be used to classify migraine. The second and third articles are phase II clinical trials which investigate the use of CGRP antagonists and a monoclonal antibody CGRP.

## Interictal increase in CGRP levels in peripheral blood as a biological marker for chronic migraine

In this study, the authors investigated the possible use of CGRP as a marker for chronic migraine. 103 female patients diagnosed with chronic migraine according to international headache society criteria were recruited. Cases reported an average of 9.5 ± 3.4 years of chronic migraine and attempted detoxification at least once for a minimum of 2 months if diagnosed with analgesic overuse migraine. The control groups consisted of 31 healthy women with no headaches and on no medications, 43 females with episodic migraine, and 14 patients with cluster headaches (13 female, 1 male).

Morning blood samples were taken from all patients on days without moderate/severe headaches. No symptomatic medications were allowed in the previous 24 h and prophylactic medications were continued. CGRP was found to be significantly higher in chronic migraine as compared to all control groups interictally. The authors subsequently analyzed the potential of CGRP as a biomarker for chronic migraine and concluded that for a CGRP concentration of 43.45 ng/ml, 90.38 % of CM, and 80.64 % of controls would be correctly classified. They also propose that it is possible to distinguish between chronic migraine and episodic migraine on the CGRP level alone.

*Comment*. Although these findings demonstrate considerable potential for CGRP levels as a disease biomarker, considerably more evidence is required before it could be used in clinical practice. Sensitivity and specificity remains poor, and its value in the clinical setting remains unclear particularly as the IHS guidelines appear to provide clear guidance for the clinician. Furthermore, the choice of control groups is limited and it would be of interest to extend these groups to include cases more representative of general clinical practice. However, the study underlines the relevance of CGRP in pathophysiology and holds promise as an objective measure of response and of treatment stratification for future therapeutic trials and also those trials specifically targeting this molecule.

Cernuda-Morollón V et al (2013) Neurology 81:1191–1196.

## BMS-927711 for the acute treatment of migraine: a double-blind, randomized, placebo controlled, dose-ranging trial

Previous studies of CGRP antagonists have been limited by early termination due to liver toxicity. This well-designed, single-dose, double-blind, randomized, multicenter evaluation of the CGRP antagonist BMS-927711 measures efficacy as an acute migraine therapy. 1026 patients were enrolled and 885 randomized to receive a single dose of placebo, 100 mg sumatriptan, or one of 6 different doses of BMS-927711 (10, 25, 75, 150, 300 or 600 mg). Exclusion criteria included a history of vascular disease and the concomitant use of drugs metabolized by CYP3A. Prophylactic migraine therapy was allowed but the use of other acute therapies was prohibited 2 days prior to randomization. A Bayesian analysis of the observed response rates allocated groups to the more effective doses of BMS-927711 as the trial progressed.

32.9 % of subjects on the 150 mg dose achieved pain freedom at 2 h post-dose. Sumatriptan and the 75, 150, and 300 mg doses of BMS-927711 were all significantly superior to placebo (<0.001, and <0.002, respectively). The 600 mg dose did not provide any additional benefits. Total migraine freedom was primarily achieved by the 75 mg dose (28.2 % of patients). The authors also explored additional efficacy endpoints such as pain relief, sustained pain relief and pain freedom over 2–48 h—all of which were achieved by sumatriptan and the 75, 150, 300 and 600 mg doses of BMS-927711.

The antagonist was overall well tolerated with a dose-related effect of nausea being the most reported adverse event. Cardiovascular side effects were only reported by patients on sumatriptan.

*Comment*. The lack of a vasoconstricting effect is a significant advantage compared to currently available 5-HT antagonists, although will need to be evaluated in patients with known ischaemic heart disease. Importantly, the authors point out that a single dose study does not provide a full side effect profile, and adverse events such as liver toxicity may only be evident with frequent use (as was the case with previous CGRP antagonist). The study was not powered to compare sumatriptan to BMS-927711, but efficacy versus placebo seemed to be comparable. Further studies are clearly needed and it will be interesting to consider whether CGRP antagonists may have a specific position in the treatment of the estimated 30 % of triptan non-responders.

Marcus R et al (2014) Cephalalgia 34(2):114–125.

## Safety, tolerability, and efficacy of TEV-48125 for preventive treatment of high-frequency episodic migraine: a multicentre, randomized, double-blind, placebo-controlled, phase 2b study

In this study, Bigal et al. report the results of two randomized, double-blind, placebo controlled phase 2b trials of a humanized monoclonal antibody targeting CGRP. This exciting new therapy raises the possibility for a new era for targeted migraine management. The study specifically included patients using preventative drugs and acute therapies, including occasional opioids, making it representative of resistant migraine and routine clinical practice.

297 patients experiencing 8–14 headache days in a month, who could comply with the electronic headache diary, were randomized in a 1:1:1 ratio of two doses of TEV 48125 (225 and 675 mg) or placebo, given subcutaneously every 28 days for 3 months. A concomitant study was performed by the same group on chronic migraineurs. By the third treatment cycle, TEV 48125 reduced the number of migraine days from baseline by 6.09 (SD 5.22) days in the 675 mg group and by 6.27 (SD 5.38) days in the 225 mg group. As in other migraine trials, a high placebo effect was present with a reduction of 3.46 migraine days. Acute drug consumption, headache days, headache hours, nausea and vomiting, photophobia and phonophobia were also significantly reduced with both doses. The monoclonal antibody was found to be safe with only mild injection site reactions reported.

*Comment*. The effectiveness and tolerability of the monoclonal antibody against CGRP, especially in patients with severe migraine, is particularly promising. Since monoclonal antibodies are too large to cross the (undisrupted) blood brain barrier, the results suggest that there is a significant peripheral therapeutic effect. Overall, the results seem very promising and reinforce the need for further trials to study the long-term safety profile of this drug, although whether the economics will make widespread use likely or possible will need to be explored.

Bigal ME et al (2015) Lancet Neurol 14:1081–1090

*Conclusion.* The neuropeptide CGRP is an intriguing target for the management of migraine, which may well change the current landscape of therapeutic options. CGRP is, however, only one of several neuropeptides involved in the trigeminocervical neuroinflammatory process (along with VIP, glutamate, etc.) and it is therefore likely that targeting this molecule will only prove effective for a limited number of patients, as indeed is the case with sumatriptan. However, it is likely to be of considerable benefit to patients who do not tolerate 5-HT antagonists. More research into the targeting of this molecule is needed, but a more complete solution for the disability relating to migraine might be possible.

